# Arthrogryposis Multiplex Congenita: Comprehensive Review from a Neuromuscular Standpoint

**DOI:** 10.3390/genes17060675

**Published:** 2026-06-09

**Authors:** Daniel Delgado Seneor, João Paulo Barile, Patrícia Marques Mendes, Marco Orsini, Eduardo Mendonça Werneck da Silva, Igor Braga Farias, Paulo de Lima Serrano, Wladimir Bocca Vieira de Rezende Pinto, Acary Souza Bulle Oliveira, Paulo Victor Sgobbi de Souza

**Affiliations:** 1 Division of Neuromuscular Diseases, Department of Neurology and Neurosurgery, Federal University of São Paulo, São Paulo 04039-060, SP, Brazil; 2 Faculty of Medicine, Iguaçu University, Nova Iguaçu 26260-045, RJ, Brazil

**Keywords:** arthrogryposis multiplex congenita, fetal akinesia deformation sequence, congenital contractures, neuromuscular disorders, neurogenetics

## Abstract

Arthrogryposis multiplex congenita (AMC) is a diverse group of conditions characterized by multiple joint contractures. Although individually rare, these disorders are estimated to affect 1 in 3000–5000 live births. Their common pathophysiological mechanism is fetal akinesia, a sustained reduction of fetal movement that may arise from intrinsic disturbances—such as central nervous system malformations, motor neuronopathies, neuropathies, neuromuscular junction defects, congenital myopathies, muscular dystrophies, or metabolic diseases—or from extrinsic factors including uterine constraint, maternal illness, infections, or toxic exposures. Reduced fetal motion leads to relatively uniform clinical manifestations, known as the fetal akinesia deformation sequence (FADS), which is characterized by craniofacial anomalies, pulmonary hypoplasia, growth restriction, and contractures. Currently, AMC is classified by clinical features, such as distal arthrogryposis or lethal congenital contracture syndromes. However, advances in molecular genetics have shown wide variability among conditions classified into the same category. Prognosis is widely variable, ranging from lethal perinatal forms to non-progressive mild conditions. This review discusses AMC etiologies from a topographic standpoint, considering the different levels of the motor system involved, by combining current clinical, genetic, and pathophysiological information.

## 1. Introduction

**Figure 1:**
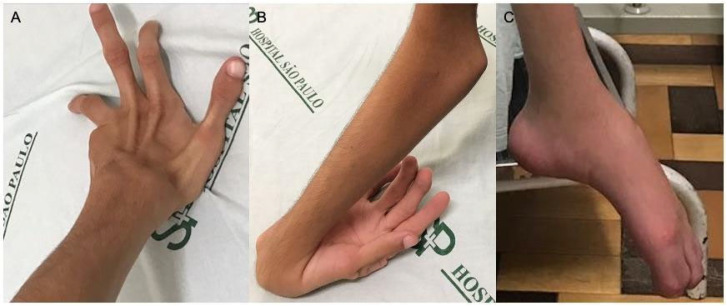
Clinical presentation of congenital joint contractures in arthrogryposis multiplex congenita (AMC). (**A**) Distal contractures of the hand with camptodactyly and limited extension of the fingers. (**B**) Wrist flexion contracture with restricted passive range of motion. (**C**) Severe equinovarus deformity of the foot, illustrating involvement of distal lower limbs. Images are from the authors’ personal clinical archive.

Arthrogryposis multiplex congenita (AMC) is fundamentally a fetal condition, as the pathogenic process leading to fixed joint positions originates in utero during critical phases of motor development. The term designates conditions presenting with congenital joint contractures in two or more body areas (Figure 1) [1]. Although often mistakenly thought to be a rare condition, the estimated incidence is 1 in 3000 to 5000 live births [2,3]. Each etiology of AMC, however, is individually rare. From a prenatal standpoint, arthrogryposis multiplex congenita (AMC) represents one of the most recognizable fetal manifestations of genetic and neuromuscular disorders.

The pathogenesis is complex and may involve multiple mechanisms, most of which result in restricted fetal movements [4,5]. The fetal akinesia deformation sequence (FADS) consists of a set of clinical findings that consistently appear in fetuses and newborns with reduced or absent movements during intrauterine life. Originally described by Pena and Shokeir [6], the sequence has since been further investigated, regarding its pathophysiologic and genetic basis [7]. Regardless of the underlying cause, the clinical expression of FADS is relatively uniform, typically including craniofacial anomalies such as hypertelorism, high nasal bridge, micrognathia, limited jaw opening, and cleft palate; joint contractures; pulmonary hypoplasia; and intrauterine growth restriction [4,8]. Nevertheless, the severity of each feature and the occurrence of additional abnormalities may vary, depending on the etiology and timing of the injury. On prenatal ultrasound, arthrogryposis becomes most evident during the mid-second trimester, typically between 18 and 24 weeks. Sonographic features include persistently fixed limb postures, clenched hands, clubfoot, joint contractures, decreased or absent fetal movements, abnormal limb extension, and occasionally polyhydramnios secondary to reduced swallowing. In this context, AMC may affect different body parts to varying degrees, reflecting the heterogeneity of neuromuscular or structural impairment that accounts for the fetal akinesia.

**Figure 2:**
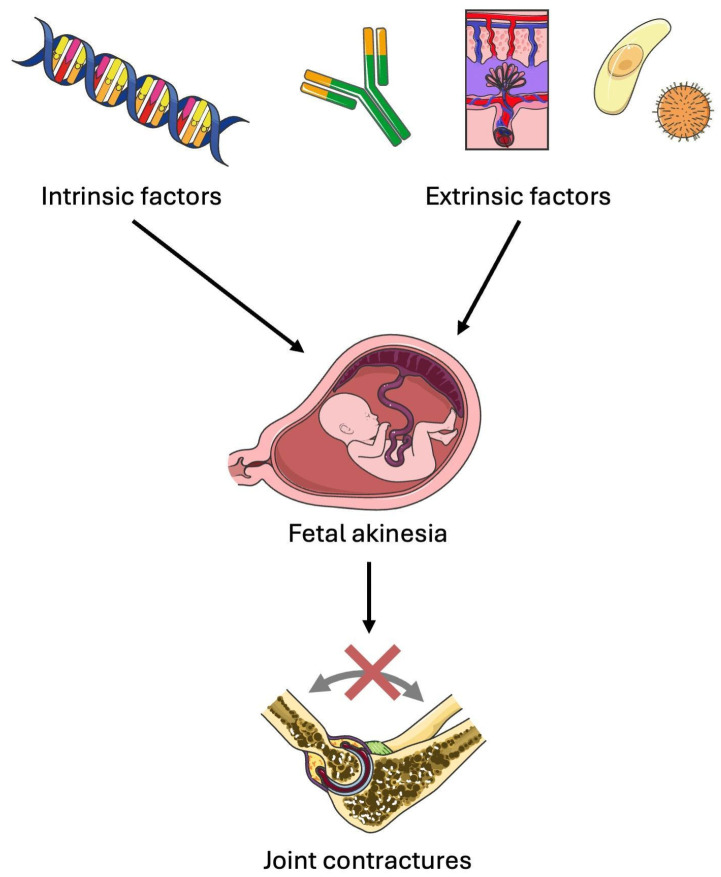
Pathophysiology of arthrogryposis. Fetal akinesia may result from both intrinsic and extrinsic factors, ultimately leading to joint contractures. Intrinsic causes are predominantly genetic in nature, whereas extrinsic factors include maternal disorders, mechanical constraints, impaired fetal vascular supply, and toxic exposures.

AMC is characterized pathophysiologically as intrinsic and extrinsic (Figure 2). Extrinsic mechanisms involve mechanical constraint, maternal illness, congenital infections, and toxic exposures (Table 1). Conversely, intrinsic mechanisms are diverse, most frequently genetic, comprising congenital motor neuronopathies, neuropathic processes, neuromuscular junction dysfunction, myopathies, hereditary metabolic diseases, connective tissue problems and CNS abnormalities (Table 2). Given the diversity in underlying causes, prognosis also varies broadly, ranging from neonatal death to mild, non-progressive, orthopaedic deformities [9]. Approximately two-thirds of affected individuals are able to live a productive life, since the most common causes (e.g., amyoplasia) have favorable outcomes in the long term.

Prior to the advent of genetic testing, the study of AMC was purely phenotypic, often aggregating different conditions together. Nevertheless, with the development of molecular diagnosis, the scientific community began to better understand the broad spectrum of this disease group. In the last few years, probably due to improvement in quality and access to molecular testing, there has been a deep expansion in our comprehension of AMC etiologies and mechanisms. The diagnostic yield of whole exome sequencing (WES) for the investigation of AMC demonstrates significant variability across clinical cohorts, achieving resolution rates that range from 42.6% up to 65.2% depending on the analytical scope and the inclusion of newly discovered or candidate genes [10,11]. The implementation of WES as a primary genomic approach reveals notable analytical superiority over targeted exome sequencing (TES) and standard gene panels, yielding a 61.1% diagnostic rate compared to 32% for TES, which effectively mitigates the limitations imposed by the extreme clinical heterogeneity of AMC and facilitates the identification of novel pathogenic loci [12]. Regarding whole genome sequencing (WGS), technical literature indicates that despite its unquestionable sensitivity advantage in capturing SNVs, indels, and copy number variations (CNVs) within non-coding regions alongside superior per-base coverage, its specific diagnostic yield for AMC cohorts has not yet been systematically evaluated.

The genes associated with AMC are involved in multiple convergent molecular pathways and gene ontologies related to fetal movement and neuromuscular development. Central nervous system forms are mainly associated with pathways involved in RNA metabolism and protein translation (*EXOSC3*, *EXOSC8*, *EXOSC9*, *TSEN54*, *RARS2*), neuronal migration and microtubule dynamics (*DCX*, *PAFAH1B1*, *TUBA1A*, *TUBB2B*, *RELN*), axonal guidance and neurodevelopment (*L1CAM*, *SOX10*), and cellular stress response pathways (*EIF2B1-5*). Motor neuronopathies predominantly involve genes related to RNA processing, ubiquitin-proteasome homeostasis, intracellular trafficking, and axonal transport, including *SMN1*, *GLE1*, *UBA1*, *DYNC1H1*, and *BICD2*. Peripheral neuropathic forms involve pathways regulating peripheral myelination, Schwann-cell development, nodal organization, and mechanotransduction, including *MPZ*, *PMP22*, *EGR2*, *GLDN*, *ADGRG6*, and *PIEZO2*. Neuromuscular junction disorders involve genes responsible for acetylcholine receptor assembly and clustering, synaptic vesicle trafficking, and endplate maintenance, such as *CHRNG*, *CHRNA1*, *RAPSN*, *MUSK*, *DOK7*, *CHAT*, and *SLC18A3*. Myopathic and distal arthrogryposis forms are linked to sarcomeric structure and excitation-contraction coupling pathways, involving genes encoding actin, myosin, troponin-tropomyosin complex proteins, and calcium-handling machinery (*ACTA1*, *MYH3*, *TPM2*, *TNNI2*, *RYR1*, *CACNA1S*, *STAC3*). Connective tissue forms are associated with extracellular matrix organization, collagen synthesis and post-translational modification pathways (*COL6A1-3*, *PLOD1*, *FKBP14*, *TNXB*, *FLNB*). Finally, metabolic forms involve pathways related to peroxisomal biogenesis, glycogen metabolism, purine metabolism, and lysosomal function (*PEX* genes, *GBE1*, *ADSL*, *GBA*). Therefore, AMC represents a final common phenotypic pathway resulting from disruption of multiple biological pathways required for normal fetal movement and musculoskeletal development.

Classifications of distal arthrogryposis, proposed by Hall [9] and Bamshad [13], comprise diseases characterized by distal joint contractures as the most prominent feature, without other remarkable systemic involvement. While they are clinically useful in phenotyping, they do not consider the different topographies affected, as many described phenotypes lack a precise genetic basis. Categorizations of lethal congenital contractures syndromes also do not consider the affected structures topographically, being numbered in order of description.

**Figure 3:**
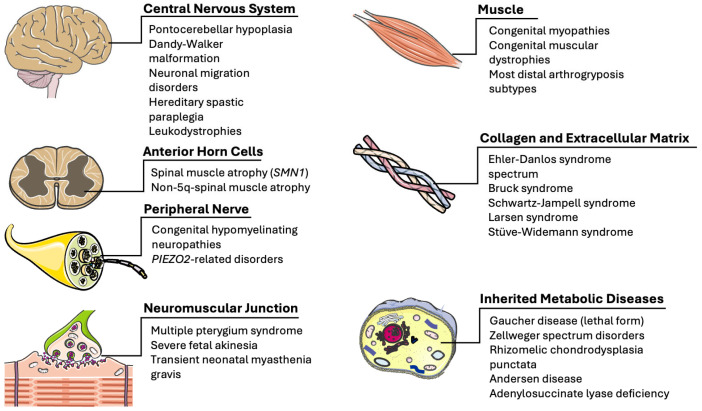
Topographic classification of arthrogryposis multiplex congenita.

**Figure 4:**
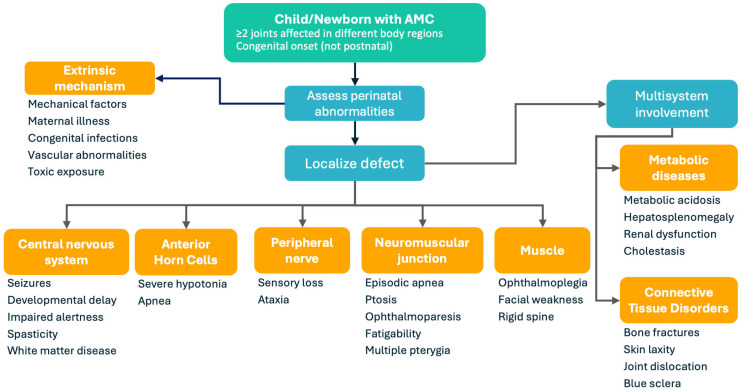
Topographical approach and respective common clinical abnormalities in patients with AMC.

**Figure 5:**
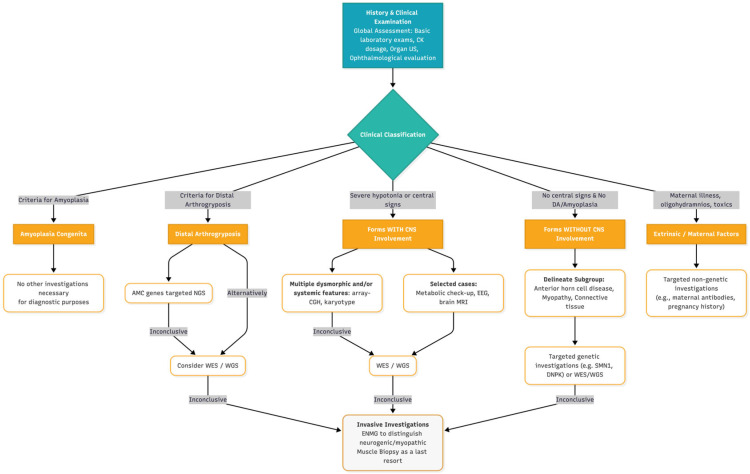
Stepwise clinical and genetic workup for the etiological assessment of children with AMC. Following an initial history and global clinical evaluation, cases are stratified into specific subgroups (amyoplasia congenita, distal arthrogryposis, intrinsic forms with or without CNS involvement, and extrinsic/maternal factors). This classification guides targeted non-genetic investigations or next-generation sequencing (NGS). As emphasized in the flowchart, whole-exome or whole-genome sequencing (WES/WGS) plays a critical role in confirming the underlying cause in inconclusive cases or as an alternative first-tier genetic test, reserving invasive investigations, such as muscle biopsy and ENMG, strictly as a last resort. Abbreviations: AMC, arthrogryposis multiplex congenita; CGH, comparative genomic hybridization; CK, creatine kinase; CNS, central nervous system; DA, distal arthrogryposis; DNPK, DN1 protein kinase; EEG, electroencephalogram; ENMG, electroneuromyography; MRI, magnetic resonance imaging; NGS, next-generation sequencing; SMN1, survival motor neuron 1; US, ultrasonography; WES, whole exome sequencing; WGS, whole genome sequencing.

Most research published on this subject focuses on genetic and/or orthopaedic aspects of AMC. However, since arthrogryposis is directly related to fetal movement and can be caused by impairment of any of the structures involved in this process, it is reasonable to adopt a topographical approach, as is commonly done with postnatal motor impairments. Understanding how distinct genetic defects affecting different levels of the motor system produce AMC is critical for accurate pre and postnatal diagnosis, counselling and prognostication. This article aims to synthesize the complexity of this condition from a neurological perspective, with a focus on genetic causes. A topographic framework is presented to clarify the main etiologies underlying congenital joint contractures, highlighting common clinical abnormalities for each topography (Figure 3 and Figure 4). Additionally, the diagnostic flowchart (Figure 5) proposes a stepwise clinical and genetic approach for the etiological assessment of patients with AMC. The workup begins with a detailed clinical history and a global assessment, enabling the stratification of cases into specific clinical subgroups: typical amyoplasia congenita, distal arthrogryposis, extrinsic/maternal factors, and complex intrinsic forms with or without central nervous system (CNS) involvement. Based on this clinical classification, investigations are tailored for each group, ranging from targeted non-genetic exams to specific next-generation sequencing (NGS) panels. A key feature of this algorithm is the emphasis on the critical role of whole-exome or whole-genome sequencing (WES/WGS), which is strongly recommended to confirm the underlying etiology when initial targeted tests return inconclusive results. By implementing this comprehensive genomic approach, invasive procedures such as electroneuromyography (ENMG) and muscle biopsies are strictly reserved as a last resort.

Importantly, the review will primarily address the clinical conditions in which AMC represents a major feature, rather than a secondary manifestation within complex multisystem disorders).

## 2. Central Nervous System Impairment

Since CNS dysfunction can cause fetal hypomobility, some of these diseases may present AMC as a clinical finding. Importantly, one should suspect that a CNS abnormality is involved in AMC pathogenesis if there is concomitant hypotonia, impaired alertness, seizures, developmental delay, and intellectual disability [14]. It is important to clarify that, in many reviews, a pathophysiological process is defined as “neuropathic” when describing any central nervous system (CNS), motor neuron disease, or nerve disorder leading to arthrogryposis multiplex congenita (AMC). For accuracy, this review uses “neuropathic” exclusively when referring to peripheral nerve impairment.

### 2.1. Central Nervous System Malformations

Primary central nervous system malformations comprise defects in brain embryological processes, such as commissure formation, posterior fossa development, and brainstem differentiation, which are directly linked to intrauterine movement. Acrocallosal syndrome (*KIF7*) is characterized by corpus callosum agenesis, facial dysmorphism, polydactyly, and inguinal hernias [15,16].

Pontocerebellar hypoplasias (PCH) are a group of autosomal recessive conditions marked by reduced pons size, cerebellar hypoplasia, and superimposed atrophy [17]. The forms related to AMC are PCH1 (*EXOSC3*, *EXOSC8*, *EXOSC9*, *VRK1*) [18], PCH4 (*TSEN54*) [19], PCH6 (*RARS2*) [20], PCH9 (*AMPD2*) [21] and PCH12 (*COASY*) [22]. The causes of multiple contractures at birth are thought to be a combination of upper motor neurons and spinal cord involvement. These PCH subtypes are associated with a poor prognosis, as most cases result in early death or profound neurological impairment.

Dandy-Walker malformation (DWM), the most common developmental abnormality of the posterior fossa, consists of agenesis or hypoplasia of the vermis, and cystic enlargement of the fourth ventricle [23]. Its genetic etiologies are diverse, some of which are associated with AMC. Aase-Smith syndrome is characterized by DWM, cleft palate, absent knuckles, and severe distal joint contractures [24]. Its genetic basis is still unknown, although an overlap with Gordon syndrome has been proposed [25]. The overlap between CNS malformations and neuropathic forms of AMC will be discussed in the section regarding neuropathic mechanisms. Pettigrew syndrome (*AP1S2*) is an X-linked disease that features DWM, congenital joint contractures, intellectual disability, basal ganglia disease and seizures [26,27]. A more complex phenotype, related to 3q microdeletions, includes DWM, blepharophimosis–ptosis–epicanthus inversus and Wisconsin syndrome [28].

Severe forms of lissencephaly (LIS) have been associated with AMC, including X-linked (*ARX*, *DCX*) [29,30] and autosomal (*PAFAH1B1*, *RELN*) forms. Miller-Dieker syndrome (del17p13.3, including *PAFAH1B1* and *YWHAE*) is a syndromic form of LIS, presenting with severe lissencephaly, facial dysmorphism and other congenital anomalies (e.g., heart, kidney and gastrointestinal defects), including joint restriction [31,32]. Tubulinopathies (*TUBA1A*, *TUBB2B*), another cause of LIS, rarely present as fetal akinesia syndrome, including joint contractures, with microlissencephaly [33,34]. A similar clinical picture has been described in patients with *BLTP1* variants, all presenting congenital joint contractures [35]. Earlier reports associate lissencephaly with AMC without detailing the genetic etiology [36,37,38,39].

Diseases involving CNS malformations along with muscle, motor neuron, nerve and/or neuromuscular junction dysfunction will be discussed in subsequent sections.

### 2.2. Non-Malformative Central Nervous System Disorders

Hereditary spastic paraplegias (HSP) are a heterogeneous group of inherited neurological disorders, characterized by degeneration of the long descending tracts. The main clinical manifestations are progressive spastic paraparesis and urinary dysfunction, but other neurological and multisystem manifestations also occur in complicated types [40]. HSP1, linked to *L1CAM*, has the characteristic feature of adducted thumbs and occasionally clubfeet, which can be considered AMC. Infants typically present with hydrocephalus, agenesis or hypogenesis of the corpus callosum and the components of the MASA syndrome (intellectual disability, aphasia, spastic paraplegia and adducted thumbs) [41,42].

Leukodystrophies are a complex group of genetic disorders that present with white matter degeneration as its main feature, some of which may present antenatally with AMC. Vanishing white matter disease (VWMD), caused by biallelic variants in *EIF2B1-5*, may rarely present prenatally with decreased fetal movements, intrauterine growth restriction, microcephaly and congenital joint contractures. There is usually multisystem involvement with cataracts, pancreatitis, hepatosplenomegaly, renal hypoplasia and ovarian dysgenesis [43].

A congenital leukoencephalopathy with complex phenotype is represented by the peripheral demyelinating neuropathy, central dysmyelinating leukodystrophy Waardenburg syndrome and Hirschsprung disease (PCWH), associated with biallelic variants in *SOX10* [44]. Interestingly, biallelic deletion of this gene has been associated with a fatal form of AMC, depigmentation, dystopia canthorum, cleft palate and cardiac malformation [45]. Oculodentodigital dysplasia is a rare genetic disorder caused by pathogenic variants in *GJA1*, characterized by distinctive facial appearance, dental anomalies, ocular abnormalities, minor distal joint contractures and digital malformations (including camptodactyly, brachydactyly, and syndactyly of the fourth and fifth fingers), often accompanied by progressive spastic paraparesis [46].

Recently, a Turkish child with pathogenic variants in *POLR3*, typically associated with hypomyelinating leukodystrophy type 7, was reported to show multiple joint contractures [47]. Another leukodystrophy recently reported to present with AMC in its severe perinatal form is *DARS2*-related leukoencephalopathy with brainstem and spinal cord involvement and lactate elevation (LBSL) [48].

## 3. Motor Neuronopathies

Motor neuronopathies (i.e., anterior horn cell disease) with prenatal onset may present with severe hypotonia, respiratory distress at birth and AMC. They may also present additional features of the fetal akinesia deformation sequence (FADS). Distal hereditary motor neuropathies (dHMN) and spinal muscular atrophies (SMA) are considered within the same group in this review.

Spinal muscular atrophy (SMA) is a group of diseases that involve degeneration of anterior horn cells in the spinal cord and motor nuclei in the brainstem, leading to progressive muscle weakness and atrophy. SMA is divided into 5q and non-5q forms based on genetic cause. 5q-SMA, the most common form, results from homozygous deletions or compound heterozygous variants in the *SMN1* gene on chromosome 5q13. Its clinical spectrum ranges from severe infantile to milder adult forms, with disease severity mainly modulated by *SMN2* copy number. Congenital SMA is the most severe form, with prenatal onset. Infants present at birth with profound hypotonia, weak or absent movements, AMC, and severe respiratory failure. Importantly, this severe presentation of SMA seems to cause multisystem abnormalities, including congenital heart defects [49,50]. Non-5q-SMAs might be classified by age of onset (congenital, infantile, juvenile or adult), weakness pattern (proximal, distal or generalized), and inheritance pattern (autosomal dominant, recessive or X-linked). Congenital forms may present with severe hypotonia, respiratory distress, and multiple contractures, whereas later onset forms usually show slowly progressive weakness, limited to proximal or distal muscle groups [51].

Among the early-onset non-5q-SMA, spinal muscular atrophy with respiratory distress type 1 (SMARD1) is a rare form of non-5q-SMA caused by variants in the *IGHMBP2* gene. The distinctive feature of the disease is diaphragmatic palsy and early respiratory distress, along with dysautonomia, severe distal weakness and joint contractures. Epilepsy and cranial nerve palsy may also occur [52,53,54]. Another phenotype within this category is dominant lower extremity-predominant spinal muscular atrophy (SMALED), characterized by proximal weakness, hypotonia and respiratory distress in severely affected individuals. *DYNC1H1* (SMALED type 1) and *BICD2* (SMALED type 2) encode the proteins that form the dynein/dynactin complex, which governs intracellular transport in all cells, including motor neurons [55,56]. Early forms of both *DYNC1H1* [53,57] and *BICD2* [58,59] have been associated with AMC. Although primarily causing motor neuronopathy, both genes have also been associated with myopathic features. Rarely, *DYNC1H1* may cause intellectual disability and neuronal migration disorders.

*GLE1* variants are associated with severe perinatal motor neuron disease in two described conditions: lethal congenital contracture syndrome 1 (LCCS1) and lethal arthrogryposis with anterior horn cell disease (LAAHD), originally described in Finnish families. LCCS1 is the most severe form, presenting with fetal hydrops, multiple contractures, facial abnormalities, pulmonary hypoplasia, muscular atrophy, and reduction in anterior horn cells. LAAHD shows a slightly milder phenotype with increased survival times, up to 20 days. Both clinical entities were described independently but appear to comprise a phenotypical spectrum of the same disease [60,61]. LCCS2 is a condition resembling LCCS1, caused by *ERBB3* variants. It has been observed in Israeli Bedouin individuals and differs from LCCS1 in ophthalmologic and urinary abnormalities, as well as absence of fetal hydrops [62].

*UBA1* (previously *UBE1*) variants are also associated with early-onset motor neuron disease, the X-linked SMA type 2 (SMAX2), which presents with congenital hypotonia, dysmorphic features, arthrogryposis, bone fractures, and death within 1 year of birth due to respiratory distress, associated neuropathologically with loss of anterior horn cells [53,63]. Variants in *TRIP4* and *ASCC1* have been reported to cause a similar phenotype, with proximal neurogenic weakness, hypotonia and bone fractures [64].

*TRPV4* variants are related to a wide variety of neuromuscular phenotypes, with substantial inter- and intrafamilial variability, including Charcot-Marie-Tooth type 2C and scapuloperoneal SMA. It has been associated with joint contractures in the context of congenital distal spinal muscular atrophy, which is characterized by distal AMC, proximal lower limb weakness, and vocal cord paralysis. Both autosomal-dominant and autosomal-recessive inheritance have been described [65,66]. 

## 4. Neuropathies

Congenital neuropathies are a heterogeneous group of rare conditions that lead to fetal nerve dysfunction, notably through defective peripheral myelination or impaired sensory mechanotransduction. They cause severe clinical consequences from early life, including perinatal hypotonia, weakness, respiratory compromise, and in some cases AMC, reflecting reduced fetal movements. These findings reveal the close link between neurodevelopmental neuropathic disturbances and the structural and functional phenotypes observed at birth.

Congenital hypomyelinating neuropathies (CHN) are rare disorders characterized by perinatal onset of progressive weakness and significantly diminished nerve conduction velocity in electrophysiological studies. The occurrence of AMC and respiratory distress is variable in these conditions. Genes in which variants cause such clinical picture include *EGR2* [67,68]; *MPZ* [69,70]; *PMP22* [71,72]; and *MTMR2* [73]. Most patients die in early infancy due to respiratory and/or infectious complications. In patients with *MPZ*-related CHN who survived early infancy, two follow-up studies indicate a non-progressive disease course, with some patients showing improvement [74,75].

*GLDN* variants have been reported to cause LCCS11, characterized by severe AMC, pulmonary hypoplasia and perinatal death in all cases. Analysis of the sciatic nerve through transmission electron microscopy in one affected fetus revealed a decrease in myelinated fibers and an increase in nodal length, which suggests that the phenotype might be explained by a neuropathic mechanism [76]. *ADGRG6* (previously named *GPR126*) genetic variants have been found to cause a similar presentation, termed LCCS9, with muscle and nerve biopsies suggesting defective myelination of the peripheral axons during fetal development [77]. Recently, a milder phenotype was described in an adult woman who presented with AMC at birth and a non-progressive, patchy, sensory-predominant neuropathy [78].

Loss of function in *LGI4* has been reported to cause severe AMC in 9 individuals with varying outcomes, including in-utero and neonatal deaths, with one 6-year-old boy alive. Common features observed were distal arthrogryposis, camptodactyly, clubfeet, and various other joint contractures and dysmorphic features [79].

A study with a large Arab family identified a homozygous variant in *ERGIC1* as the cause of neuropathic arthrogryposis multiplex congenita with a mild clinical course. Patients exhibited flexion contractures, muscle hypotrophy, and weakness. Neurological examination and neurophysiological studies were consistent with a neuropathic process [80]. Subsequently, complete biallelic loss-of-function caused by the promoter and first exon deletion in the same gene was reported to cause isolated mild AMC [81].

Among the etiologies of distal arthrogryposes, the *PIEZO2* gene stands out for its important role in mechanotransduction, involved in vibration sense and proprioception [82]. Gain-of-function in this gene is responsible for four well-described phenotypes: Marden-Walker syndrome, Gordon syndrome (distal arthrogryposis type 3), distal arthrogryposis type 5A, and distal arthrogryposis with impaired proprioception and touch. Marden-Walker syndrome is the most severe, characterized by blepharophimosis, intellectual disability, and hindbrain malformations such as Dandy-Walker syndrome [83]. Gordon syndrome presents with distal joint contractures and cleft palate, while intelligence remains normal [84]. Distal arthrogryposis type 5A features ophthalmoplegia, eyelid ptosis, and may also include restrictive lung disease due to increased chest-wall muscle tone [85]. A similar phenotype has been described in individuals with *ECEL1* variants [86,87]. Conversely, *PIEZO2* loss-of-function causes distal arthrogryposis with impaired proprioception and touch (DAIPT), characterized by remarkable sensory impairment, distal AMC, severe scoliosis, sensory ataxia, and short stature [88,89,90,91]. More severe forms have also been described, with neonatal respiratory distress, feeding difficulties, and hypotonia [92].

## 5. Disorders of the Neuromuscular Junction

The neuromuscular junction is a key structure that transduces electrical activity from the distal nerve terminal of the motor neuron to post-junctional muscle membrane, ultimately generating muscle contraction. Severe congenital defects in components of this complex structure might lead to severe fetal akinesia and, ultimately, AMC [93,94]. Multiple processes might be involved in this clinical picture: presynaptic neurotransmitter synthesis and release, vesicle trafficking, acetylcholine synthesis and degradation, acetylcholine receptor binding and concentration, extracellular matrix stability, and appropriate endplate structure [94].

The most characteristic descriptions of AMC related to neuromuscular junction disorders are the multiple pterygium syndromes, although other pathophysiological mechanisms and topographies (notably myopathies) have been described. The non-lethal phenotype, extensively characterized as Escobar syndrome, shows multiple pterygia, joint contractures, and distinctive facial features [95]. Subsequently, the syndrome was genetically linked to variants in *CHRNG* [96,97]. This gene encodes the γ subunit of the acetylcholine receptor at the neuromuscular junction and its deficiency leads to reduced fetal movements. After 33 weeks of gestation, the γ subunit is replaced by the ε subunit, which accounts for the postnatal absence of myasthenic symptoms and the stable postnatal phenotype [98]. Conversely, lethal phenotypes caused null variants in *CHRNA1* and *CHRNG* present with severe FADS, AMC, fetal hydrops and spontaneous abortion or early intrauterine death [93].

MuSK contributes to formation, maturation, and maintenance of the endplate, and its deficiency is extremely rare [94]. *MUSK* variants have been reported to cause pulmonary hypoplasia, facial dysmorphism, reduced muscle bulk, and widespread joint contractures, a phenotype categorized as FADS1. Pterygia and cleft palate were absent [99,100]. Rapsyn (*RAPSN*) is an important protein that anchors and clusters acetylcholine receptors at the neuromuscular junction, ensuring proper synaptic transmission. Its deficiency causes a severe form of congenital myasthenic syndrome, often with AMC, ptosis, ophthalmoparesis and early respiratory distress. The clinical course throughout life is variable and patients usually respond to pyridostigmine [101,102]. The allelic condition is FADS2, a lethal form of AMC, that clinically resembles FADS1 [94]. Malfunction of *NUP88*, a protein that interacts with Rapsyn, has been reported to cause a novel form of FADS [103]. *DOK7* plays a central role in endplate maintenance, and has been associated with severe lethal FADS with AMC, categorized as FADS3 [104,105,106,107].

Genetic variants in genes that encode presynaptic components, including *CHAT*, *SNAP25B* and *SLC18A3* have been described to cause AMC. *CHAT* mutations are well described, causing early-onset congenital myasthenic syndrome, often presenting with neonatal apneic crises (sometimes stress-triggered), and ptosis without ophthalmoplegia. More severe cases show marked hypotonia, and some cases present with AMC [94,108,109]. To date, two patients with *SNAP25B* variants have been described, both presenting intrauterine hypotonia, neonatal respiratory distress, delayed psychomotor development, AMC, fatigable muscle weakness, and ptosis. Both showed CNS impairment, with ataxia and early epilepsy [110,111]. A patient with variants in *SLC18A3*, encoding the vesicular acetylcholine transporter, has been reported to manifest early myasthenic syndrome with AMC and neonatal cyanosis [112].

Transient neonatal myasthenia gravis (TNMG) in newborns of mothers with myasthenia gravis (MG) is an important extrinsic cause of AMC resulting from neuromuscular junction dysfunction. It results from transplacental transfer of maternal immunoglobulin G antibodies targeting acetylcholine receptors (AChR). These antibodies impair neuromuscular transmission in the fetus by the same immunopathogenic mechanisms observed in MG. The presence of antibodies with affinity for the fetal isoform of AChR has been related to the condition [113,114]. Usually, TNMG presents within the first hours to days after birth, manifesting as hypotonia, weak sucking, poor swallowing, respiratory distress, and occasionally generalized weakness [115]. However, some patients show a more severe and irreversible presentation, including AMC, facial diplegia, and skeletal deformities. In the most severe cases, reduced intrauterine movement leads to FADS, which is often associated with pulmonary hypoplasia and high neonatal mortality [115,116,117]. A transient neonatal myasthenic syndrome has been reported in the context of Lambert-Eaton myasthenic syndrome [118]. However, to date, no cases of congenital joint contractures have been reported. Although TNMG is transient in most infants and resolves within weeks as maternal antibodies are cleared, AMC represents an antenatal and often irreversible complication linked to sustained fetal paralysis during critical developmental periods.

## 6. Myopathies

Because muscle development is essential for fetal intrauterine movement, factors that impair this process are a major cause of AMC. The main disease groups that compromise muscle tissue at fetal stages and can cause AMC are amyoplasia, congenital myopathies and congenital muscular dystrophies. Causes of distal arthrogryposis forms with impaired muscle development are discussed together with congenital myopathies, as the pathophysiological mechanism and structural abnormalities are similar.

### 6.1. Amyoplasia

The most frequent cause of arthrogryposis is amyoplasia, representing approximately one-third of all cases. This condition is sporadic and consists of the absence of muscle development, resulting in congenital joint contractures, fibrofatty replacement of muscle tissue, and reduced fetal movement, with normal intelligence and sensation preserved [9,119,120,121]. Most patients present with involvement of all four limbs (more than 50%), but presentations limited to a single limb or restricted to either the upper or lower limbs also occur. Although the exact cause remains unclear, current evidence suggests that vascular compromise between the 7th and 12th gestational weeks is a central factor in its pathogenesis. This is supported by the frequent association with gastrointestinal anomalies of apparent vascular etiology, midline hemangiomas, abdominal wall defects, digit reductions, and other findings compatible with impaired intrauterine blood flow. Amyoplasia carries no evidence of genetic inheritance, with absent recurrence within families and no consistent effects of parental ages. Regardless of the severity of orthopedic involvement, long-term prognosis for affected individuals is favorable, with appropriate surgical, orthopedic, and rehabilitative care [9,120].

### 6.2. Congenital Myopathies

Congenital myopathies are a broad group of neuromuscular disorders, and AMC is one of their most prominent manifestations in prenatal-onset forms. In severe neonatal cases, reduced fetal movements are often reported and play a central role in the development of joint contractures. At birth, severely affected infants commonly present with profound hypotonia, respiratory insufficiency, difficulties in feeding, and facial hypomimia. The clinical course is usually stable or progresses slowly, with hypotonia and muscle atrophy persisting into adulthood. Skeletal complications, such as hip dysplasia, scoliosis, and rigid spine, may emerge as patients grow. A widely used classification to congenital myopathies is based on histopathological features. However, this approach is limited by the fact that different genetic mutations may be responsible for multiple histopathological patterns, while each pattern may arise from various genetic mutations.

Variants in genes encoding sarcomeric proteins represent an important cause of congenital myopathies frequently associated with AMC. Disruption of actin filaments, as seen in *ACTA1* and *ACTC1* variants, compromises the structural backbone of the thin filament and reduces contractile capacity. *ACTA1* (α-Skeletal Actin) has been associated with a severe form of congenital myopathy, with facial weakness, feeding and swallowing difficulties, and respiratory insufficiency, without cardiac involvement [122,123]. *ACTC1*, which encodes α-cardiac actin 1 in cardiac and fetal skeletal muscle, has been described to cause multiple congenital contractures, neck pterygia, scoliosis, and congenital heart defects/cardiomyopathy [122]. Variants in thin filament stabilizers *KLHL40* and *LMOD3* cause rare, often severe, subtypes of nemaline myopathy. The clinical features comprise early-onset severe hypotonia, ophthalmoplegia, respiratory insufficiency, feeding difficulties, and AMC [124,125,126,127].

Similarly, pathogenic variants in myosin heavy chains (MyHC) directly impair muscle force generation, playing a critical role during early developmental stages [128]. *MYH2* causes severe myopathy, with respiratory distress, hypotonia, and myopathic facial appearance with ophthalmoplegia and inability to swallow [129,130]. *MYH3*, which encodes the embryonic isoform of MyHC, is primarily associated with distal arthrogryposis, most notably Freeman-Sheldon (DA2A) and Sheldon-Hall syndromes (DA2B) [131,132]. In Freeman-Sheldon syndrome, craniofacial abnormalities such as microstomia, “whistling face”, micrognathia, and deep chin dimpling are prominent. Sheldon-Hall syndrome presents with milder facial involvement but similar distal contractures. Beyond distal arthrogryposis (DA), pathogenic variants in *MYH3* are also associated with contractures, pterygia and spondylocarpotarsal fusion syndrome 1, which is characterized not only by joint contractures but also by multiple fusions of the vertebrae, carpal, and tarsal bones [133,134]. Finally, trismus-camptodactyly (DA7) is a condition caused by pathogenic variants in *MYH8* and is classified as a distal arthrogryposis. This condition is characterized by limited mouth opening, pseudocamptodactyly, which is defined by involuntary flexion of the fingers triggered by wrist extension but not by flexion [135,136].

*TNNI2*, *TNNT3*, and *TPM2* encode proteins that constitute the troponin-tropomyosin complex, which is responsible for regulating skeletal muscle contraction through control of actin-myosin interaction in response to calcium signaling. *MYBPC1* and *MYBPC2* encode myosin-binding protein C, which stabilizes the thick filaments of the sarcomere. *MYLPF* encodes the myosin regulatory light chain that modulates the cross-bridge cycling and generation of muscle strength. Dysfunction of these mechanisms leads to sarcomere dysfunction and impaired contractile function. Clinically, variants in TNNI2, *TPM2*, *MYBPC1*, *MYBPC2*, and *MYLPF* have been associated with distal arthrogryposis type 1 (DA1) [9,137], whereas variants in *TNNT3*, *TNNI2*, and *TPM2* might cause Sheldon-Hall syndrome (DA2B) [9,138].

Titin (TTN) and nebulin (NEB) are giant sarcomeric proteins that maintain muscle integrity. Titin provides elasticity, alignment, scaffolding, and mechanosensing, while nebulin defines actin filament length, stabilizes thin filaments, and regulates contraction efficiency [139]. Congenital titinopathies present with reduced fetal movements, distal joint contractures, and early feeding and/or respiratory difficulties. Muscle weakness is usually proximal, symmetrical, and of mild to moderate severity, accompanied by hypotonia and muscle atrophy. The clinical spectrum is broad: while some patients achieve ambulation and follow a slowly progressive course, others develop FADS, severe AMC, persistent respiratory, feeding impairment, and terminal heart failure. Notably, AMC has been reported in 43% of patients with congenital titinopathy, and up to 50% also present with congenital heart defects or cardiomyopathy [140,141]. Variants in *NEB* can manifest with AMC in severe fetal and neonatal cases, presenting reduced fetal movement, multiple joint contractures, polyhydramnios, dysmorphic features, and respiratory failure leading to early death. Cardiac involvement is usually absent, with normal myocardial histology, and occasional findings are not consistent [142,143,144].

Defects in excitation–contraction coupling represent a critical mechanism underlying AMC. Mutations in *RYR1* can present with arthrogryposis, as shown in a series of 12 neonatal-onset patients where reduced fetal movements, contractures, scoliosis, and respiratory impairment were common, with two early deaths [145]. In a pediatric cohort, 18% of *RYR1*-related congenital myopathy patients showed congenital joint contractures [146]. Similarly, biallelic *CACNA1S* variants cause a severe congenital myopathy with fetal akinesia, hypotonia, respiratory failure, cleft palate, and congenital contractures [147,148]. Missense biallelic variants in *STAC3*, first described in Native American myopathy, are related to hypotonia, respiratory failure, and ophthalmoplegia, with AMC present in 38% of patients [149].

Variants in genes involved in membrane organization and cytoskeletal dynamics, such as *DST* and *FILIP1*, have been implicated in AMC, showing how disruption of these pathways can lead to reduced fetal movement and congenital contractures. Variants in *DST* have been reported in lethal congenital phenotypes with generalized contractures [150]. More recently, *FILIP1* loss-of-function variants were identified as a cause of AMC, with affected infants showing decreased fetal movements, generalized contractures, scoliosis, early respiratory involvement and microcephaly [151].

### 6.3. Congenital Muscular Dystrophies

Congenital muscular dystrophies (CMDs) are inherited disorders primarily affecting skeletal muscle. They are characterized by hypotonia and weakness present before patients achieve independent ambulation, delayed or arrested gross motor milestone attainment, with dystrophic muscle pathology. Since they are characterized by severe structural muscle abnormalities, AMC is relatively common. Pathophysiologically, CMD subtypes most associated with AMC are those related to defects in structural proteins of the basement membrane or extracellular matrix, abnormal α-dystroglycan glycosylation defects, and disrupted nuclear envelope. Although severity varies widely, this section will focus on severe presentations, responsible for causing AMC.

The two main types of CMD affecting structural proteins of the basement membrane or extracellular matrix of muscle fibers are *LAMA2*- and *COL6*-related disorders. Neonates with LAMA2-related disorder usually present with severe hypotonia, respiratory distress and rarely achieve trunk control. CNS involvement is common, with white matter hyperintensity and epilepsy in 30% of cases. AMC is a common feature, while cardiomyopathy is exceptional [152]. Variants in *COL6A1*, *COL6A2* and *COL6A3* cause a severe phenotype known as Ullrich CMD, which is characterized by congenital joint contractures, hypotonia, spinal rigidity, and diaphragmatic weakness. Characteristic clinical features include hyperextensibility of joints, hip dislocation, keloid scarring and follicular hyperkeratosis [153].

α-Dystroglycanopathy is a group of disorders associated with multiple genes, in which protein structure or, more commonly, the O-mannosylation of α-DG is deficient. α-DG’s main role is to anchor the myocyte cytoskeleton to the extracellular matrix and basal lamina dystrophin-glycoprotein complex. The three main severe forms of dystroglycanopathies that may cause AMC are, in decreasing order of severity, Walker Warburg syndrome (WWS), muscle-eye-brain (MEB) disease and Fukuyama congenital muscular dystrophy (FCMD). Walker–Warburg syndrome is the most severe one, characterized by early fetal onset with profound brain malformations, severe ocular anomalies, generalized hypotonia, and death usually within the first years of life. The presence of AMC varies among reported cases [154,155]. MEB and FCMD are milder than WWS and present with hypotonia, less severe brain structure abnormalities, developmental delay, ocular involvement, and survival typically extending into the second or third decade of life [156].

Nuclear envelopathies are a group of genetic disorders characterized by defects in the nuclear envelope and its connection to the cytoskeleton. Notably, those caused by *LMNA* and *SYNE1* variants, which are associated with Emery-Dreifuss muscular dystrophy, have been reported to cause severe neonatal phenotypes with marked hypotonia and AMC [157,158].

Congenital myotonic dystrophy, the most severe form of myotonic dystrophy type 1, is caused by unstable CTG repeat expansion in the *DMPK* gene. This disorder presents the phenomenon of anticipation, with increasingly severe disease in successive generations, often manifesting as the congenital form when maternally inherited. Expanded repeats cause a toxic RNA gain-of-function, leading to spliceopathy that disrupts muscle development and function. Clinically, CDM is frequently associated with arthrogryposis, polyhydramnios, reduced fetal movements, and preterm delivery. Neonates present with profound hypotonia, ptosis, craniofacial dysmorphism, respiratory and feeding difficulties. Unlike in adults, cardiac involvement is usually absent or, when present, limited to conduction abnormalities and valve defects [159].

## 7. Hereditary Metabolic Diseases

A wide range of hereditary metabolic diseases may present with AMC, most often due to impairment of the central nervous system and/or muscle tissue. AMC tends to occur in the most severe forms of these conditions, in which early profound fetal hypokinesia is a prominent feature.

Lysosomal storage diseases are a heterogeneous group of inherited metabolic disorders characterized by the abnormal accumulation of substrates within lysosomes, leading to multisystem dysfunction. In these disorders, AMC results from infiltration of connective and bone tissues by storage material and from associated neurological dysfunction. The perinatal-lethal form of Gaucher disease (*GBA1*) has been associated with AMC, frequently accompanied by hepatosplenomegaly, generalized hypotonia, petechiae, ichthyosis, and the presence of a collodion membrane [160,161]. The perinatal form of multiple sulfatase deficiency (*SUMF1*) has also been associated with joint contractures, presenting with coarse facial features, dysostosis multiplex, hip dysplasia, ichthyosis, hepatomegaly and, less commonly, hydrocephalus or hydrops fetalis [161,162,163,164]. Although mucopolysaccharidoses are characterized by severe and progressive skeletal involvement, they rarely present with AMC, which is usually restricted to the feet.

Peroxisomal disorders are an important cause of AMC, since peroxisome dysfunction has important roles in myelination, neuronal migration and axonal transport. AMC might be present in Zellweger syndrome (*PEX1*), which is characterized by progressive, severe neurologic dysfunction, craniofacial abnormalities, and liver dysfunction [165]. Another key disorder in this group is rhizomelic chondrodysplasia punctata (RCDP), of which five molecularly distinct types have been described. This condition presents with a range of skeletal abnormalities, most notably rhizomelic shortening and punctate calcifications. It is also associated with distinctive facial features, intellectual disability, and congenital cataracts. All types of RCDP (caused by variants in *PEX7*, *GNPAT* and *AGPS*) are inherited in an autosomal recessive manner and can be associated with congenital joint contractures [166,167].

Congenital forms of metabolic myopathies might cause FADS, leading to arthrogryposis. Andersen disease (glycogen storage disease type IV—*GBE1*), characterized by glycogen branching enzyme deficiency, has been related to AMC. The most severe expression is the fatal perinatal form, characterized by hydrops fetalis and FADS, including AMC [168,169]. A less severe, non-lethal neonatal presentation has also been described, with clinical features that include hypotonia, multiple joint contractures, variable central nervous system involvement, early dilated cardiomyopathy, and respiratory failure [169,170,171].

Adenylosuccinate lyase deficiency is a metabolic defect of purine synthesis, resulting in accumulation of toxic metabolites [172]. The clinical spectrum ranges from severe neonatal forms to milder phenotypes with later onset. Prenatal manifestation has been associated with intrauterine growth restriction, hypotonia, respiratory failure, and AMC. Postnatally, patients often present with developmental delay, epilepsy, autistic features, and progressive neurologic deterioration [172,173].

Arthrogryposis, renal dysfunction, and cholestasis (ARC) is a severe multisystem disorder caused by pathogenic variants in *VPS33B*, *VIPAS39*, or *PLOD3*, which lead to impaired intracellular trafficking and abnormal collagen modification. Loss of epithelial polarity causes abnormal localization of bile transporters in hepatocytes, generating neonatal cholestasis. Abnormal collagen disrupts renal tubules structure, resulting in renal failure and proteinuria, and muscle structure, leading to fetal akinesia and AMC. Other manifestations include ichthyosis, platelet dysfunction, recurrent infections, and early mortality, usually within the two first years of life [153].

Other hereditary metabolic diseases with severe central nervous system involvement may present with AMC, but joint contractures are not a defining phenotypic feature.

## 8. Hereditary Connective Tissue Disorders

Hereditary connective tissue disorders (HCTDs), caused by defects in collagen or other extracellular matrix components, can lead to AMC by altering collagen synthesis, fibrillogenesis, or remodeling. A recent review emphasizes collagen-related mechanisms as a distinct molecular pathway underlying arthrogryposis [153]. Of note, *COL6*-related disorders lie at the interface between connective tissue disorders and myopathies. However, because their clinical phenotype aligns more closely with a muscular dystrophy, they are discussed in Section 6.3.

Although present only in the CNS in adults, Collagen XXV plays an important role during fetal limb development [174]. A report correlated recessive variants in *COL25A1*, previously associated with isolated congenital ocular cranial denervation, with AMC in 5 individuals. Most affected patients presented this neuro-ophthalmological phenotype and respiratory difficulties, with additional features such as vocal cord paralysis and cognitive impairment observed in some cases [175].

Ehlers–Danlos syndromes (EDS) are a group of disorders characterized by hypermobile joints, hyperextensible skin, and fragile tissues. They arise from abnormalities in the synthesis, structure, or post-translational modification of collagen or associated proteins. The kyphoscoliotic type (kEDS), caused by variants in *PLOD1* or *FKBP14*, presents with congenital hypotonia, progressive scoliosis, and joint contractures, which might be present at birth. The musculocontractural type (mcEDS), linked to *CHST14* or *DSE*, is characterized by congenital contractures of fingers, elbows, knees, and talipes equinovarus, with distinctive craniofacial features and fragile skin. The spondylodysplastic type (spEDS), associated with variants in *B4GALT7*, *B3GALT6*, or *SLC39A13*, shows short stature, hypotonia, and congenital joint stiffness or contractures. Less frequently, classical-like EDS type 2 (clEDS2), caused by variants in *TNXB*, may also present with congenital joint limitations [176].

Defects in the post-translational modification of collagen can result in more complex phenotypes. Bruck syndrome types 1 and 2 (associated with *PLOD2* and *FKBP10*) are characterized by recurrent fractures, congenital contractures, skeletal deformities, blue sclera, and hearing loss [177]. Variants in *PLOD3* cause a complex connective tissue disorder with skin laxity, vascular fragility, facial dysmorphism, and AMC [178].

Camptodactyly–arthropathy–coxa vara–pericarditis (CACP) syndrome is caused by variants in *PRG4*, which encodes lubricin, a glycoprotein that reduces friction in synovial joints. It leads to noninflammatory arthropathy and joint contractures that may be present at birth. Other features include coxa vara deformity and recurrent pericarditis or pleuritis, due to defective lubricin in serosal membranes [179].

In Schwartz–Jampel syndrome, caused by loss-of-function variants in *HSPG2*, defective perlecan disrupts the basement membrane, leading to myotonia, short stature, blepharophimosis, and generalized joint stiffness beginning in early gestation [180]. Filamins are large actin-binding proteins that cross-link actin filaments and anchor them to membrane receptors and extracellular matrix components, playing essential roles in skeletal morphogenesis. Larsen syndrome, linked to variants in FLNB, presents with osteochondrodysplasia, large-joint dislocations, hearing loss, cleft palate, short stature and AMC [181].

Stüve–Wiedemann syndrome, caused by biallelic *LIFR* mutations, involves defective cytokine signaling via the JAK/STAT3 pathway, essential for connective tissue homeostasis. The resulting phenotype closely mimics that of structural HCTDs, with affected neonates presenting congenital joint contractures, bowed long bones, facial dysmorphism, and episodes of hyperthermia or respiratory instability [182] (Romeo Bertola et al., 2016).

## 9. Conclusions

There is no doubt that AMC must be included as a core component of good clinical practice in both medical and surgical specialties. The wide range of affected topographies and associated dysfunctions reflects the complex genetic background that underlies this condition, particularly in neuromuscular disorders. The proposed topographic approach offers a clearer understanding of the diverse mechanisms involved in congenital contractures and supports more accurate prenatal and postnatal diagnosis, counselling, and prognostication. This review aims to provide a broader and more integrated perspective on the main differential diagnoses related to AMC, emphasizing genetic and pathophysiological mechanisms underlying each clinical entity.

Although this work provides a comprehensive topographical framework for understanding the neuromuscular and genetic aspects of arthrogryposis multiplex congenita, it has some limitations. First, a narrative review rather than a systematic one, the literature selection is susceptible to bias, and a systematic statistical appraisal of all reported genetic variants was not performed. Second, because our primary focus was on the intrinsic neurological and muscular underpinnings of fetal akinesia, purely mechanical and extrinsic factors may be underrepresented. Furthermore, while we categorized the genetic etiologies based on our topographic approach, we did not detail the specific molecular pathways and gene ontologies for all the implicated genes. Finally, the field of neurogenetics is rapidly advancing, and the exact list of genes associated with AMC are subject to ongoing evolution and discovery.

## Figures and Tables

**Table 1:** Main extrinsic causes of congenital multiple distal arthrogryposis.

Category	Causes
Mechanical factors	Multiple pregnancy Uterine malformations (e.g., bicornuate or septate uterus) Uterine fibroids
Maternal illnesses	Maternal infections (e.g., Zika virus, Coxsackie, CMV) Autoimmune diseases (e.g., myasthenia gravis, lupus) Poorly controlled maternal diabetes
Congenital infections	Zika virus, CMV, toxoplasmosis, rubella
Placental and vascular factors	Placental infarction Uteroplacental insufficiency Fetal vascular abnormalities
Environmental factors	Exposure to toxins or pesticides Drugs (e.g., neuromuscular blockers, misoprostol, alcohol, cocaine)

**Table 2:** Main intrinsic causes of congenital multiple distal arthrogryposis. PCH—Pontocerebellar hypoplasia; HSP1—Hereditary spastic paraplegia type 1; PCWH—peripheral demyelinating neuropathy, central dysmyelinating leukodystrophy, Waardenburg syndrome, and Hirschsprung disease; SMA—Spinal muscular atrophy; DA—Distal arthrogryposis; FADS—Fetal akinesia deformation sequence; CACP—Camptodactyly–arthropathy–coxa vara–pericarditis syndrome.

Category	Condition/Syndrome	Gene(s)	Remarkable Features
CNS malformations	Pontocerebellar hypoplasias	PCH1 (*EXOSC3*, *EXOSC8*, *EXOSC9*, *VRK1*) PCH4 (*TSEN54*) PCH6 (*RARS2*) PCH9 (*AMPD2*) PCH12 (*COASY*)	Reduced pons size and cerebellar hypoplasia.
	Dandy-Walker malformation	Unknown–genetic and environmental	Vermis hypoplasia and cystic enlargement of the fourth ventricle
	Lissencephaly	*ARX*; *DCX*, *PAFAH1B1*; *RELN*; *PAFAH1B1*; *YWHAE*; *TUBA1A*; *TUBB2B*; *BLTP1*	Progressive demyelination; ataxia, weakness
Non-malformative CNS disorders	HSP1	*L1CAM*	Adducted thumbs
	Vanishing white matter disease	*EIF2B1-5*	Microcephaly, multiple organ involvement
	PCWH	*SOX10*	Pigmentary abnormalities, hearing loss, Hirschprung disease
	Oculodentodigital dysplasia	*GJA1*	Dental and ocular abnormalities
Motor neuronopathies Motor neuronopathies (anterior horn cell disease)	Spinal muscular atrophy (congenital)	*SMN1*	Severe hypotonia, respiratory failure
	Non-5q-SMA	*IGHMBP2*; *DYNC1H*; *BICD2*; *GLE1*; *ERBB3*; *UBA1*; *TRIP4*; *ASCC1*; *TRPV4*	Variable
Neuropathies	Congenital hypomyelinating neuropathies	*EGR2*; *MPZ*; *PMP22*; *MTMR2*; *GLDN*; *ADGRG6*; *LGI4*; *ERGIC1*	Fetal akinesia, nerve hypomyelination
	Distal arthrogryposis with impaired proprioception and touch	*PIEZO2*	Severely impaired proprioception, scoliosis
	Gordon syndrome (DA type 3)	*PIEZO2*	Cleft palate, broad joint involvement
Neuromuscular junction abnormalities	Multiple pterygium syndrome (Escobar type)	*CHRNG*	Multiple pterygia, distinctive facial features
	Severe FADS	*CHRNA1*; *CHRNG*; *RAPSN*; *MUSK*; *DOK7*; *NUP88*; *CHAT*; *SNAP25B*; *SCL18A3*	
	Transient neonatal myasthenia gravis	Sporadic–mothers with myasthenia gravis	Hypotonia, weak sucking, respiratory distress
Myopathies	Congenital myopathies	*RYR1*; *CACNA1S*, *STAC3*, *MTM1*; *DNM2*; *SPN1*; *ACTA1*; *ACTC1*; *TPM2*; *KLHL40*; *LMOD3*; *TNNT1*; *MYH2*; *TTN*; *NEB*	Generalized muscle weakness, hypotonia, axial involvement. Cardiomyopathy (ACTC1)
	Congenital muscular dystrophies	*LAMA2*; *LMNA*; *COL6A1-3*	Hypotonia, axial contractures (LMNA, EDM), CNS involvement (LAMA2), hyperextensibility (COL6A1, 2, 3)
	Congenital myotonic dystrophy	*DMPK*	Myotonia
	Distal arthrogryposis 1A	*TNNI2*; *TPM2*; *MYBPC1*; *MYBPC2*; *MYLPF*	Pure distal AMC
	Freeman–Sheldon syndrome (DA 2A)	*MYH3*	‘Whistling face’ with ulnar deviation of hands
	Sheldon–Hall syndrome (DA 2B)	*MYH3*; *TNNI2*; *TNNT3*; *TPM2*	Camptodactyly, triangular facies
	Trismus-camptodactyly	*MYH8*	Jaw contracture
Hereditary connective tissue disorders	Ehlers–Danlos syndrome spectrum	*PLOD1*; *FKBP14*; *CHST14*; *DSE*; *B4GALT7*; *B3GALT6*; *SLC39A13*; *TNXB*	Fragile skin, scoliosis, short stature
	Bruck syndrome	*FKBP10*; *PLOD2*	Brittle bones, blue sclera
	CACP syndrome	*PRG4*	Noninflammatory arthropathy, pericarditis
	PLOD3-related disorder	*PLOD3*	Skin laxity, vascular fragility,
	Schwartz–Jampel syndrome	*HSPG2*	Myotonia, short stature, blepharophimosis
	Larsen syndrome	*FLNB*	Osteochondrodysplasia, joint dislocations, hearing loss, cleft palate
	Stüve–Wiedemann syndrome	*LIFR*	Skeletal dysplasia, hyperthermia, respiratory instability
Hereditary Metabolic Diseases	Gaucher disease (perinatal lethal)	*GBA*	Hydrops, unusual fat, hepatosplenomegaly
	Zellweger spectrum disorders	*PEX1*; *PEX6*; *PEX10*	Peroxisome biogenesis defect; neuronal migration abnormalities
	Rhizomelic chondrodysplasia punctata	*PEX7*, *GNPAT*, *AGPS*	Rhizomelic shortening, congenital cataracts
	Andersen disease	*GBE1*	Hypotonia, cardiomyopathy, respiratory failure
	Adenylosuccinate lyase deficiency	*ADSL*	Hypotonia, respiratory failure, CNS involvement

## Data Availability

No new data were created or analyzed in this study. Data sharing is not applicable to this article.

## Abbreviations

The following abbreviations are used in this manuscript:

AMC	Arthrogryposis Multiplex Congenita
ARC	Arthrogryposis–Renal Dysfunction–Cholestasis Syndrome
CMD	Congenital Muscular Dystrophy
CNS	Central Nervous System
EDS	Ehlers–Danlos Syndrome
FADS	Fetal Akinesia Deformation Sequence
FCMD	Fukuyama Congenital Muscular Dystrophy
HSP	Hereditary Spastic Paraplegia
LCCS	Lethal Congenital Contracture Syndrome
PCWH	Peripheral Demyelinating Neuropathy, Central Dysmyelinating Leukodystrophy, Waardenburg Syndrome, and Hirschsprung Disease
SMA	Spinal Muscular Atrophy
SMARD1	Spinal Muscular Atrophy with Respiratory Distress Type 1
SMALED	Lower-Extremity–Predominant Spinal Muscular Atrophy
TNMG	Transient Neonatal Myasthenia Gravis
VWMD	Vanishing White Matter Disease
WWS	Walker–Warburg Syndrome
